# Cognitive Impairment Associated with Chemotherapy: Neuroimmunological Interactions, Gut–Brain Axis, and Therapeutic Approaches

**DOI:** 10.3390/brainsci16070765

**Published:** 2026-07-21

**Authors:** Beatriz Alejandra Llanes-Cervantes, José Alfonso Cruz-Ramos, María Esthela Barón-Cárdenas, Paola Montserrat Zepeda-Olmos, Sandra López-Verdín, Jennifer Mariana Vargas-López, Emmanuel de la Mora-Jiménez

**Affiliations:** 1 Licenciatura en Nutrición, Centro Universitario de Ciencias de la Salud, Universidad de Guadalajara, Guadalajara 44340, Mexico; 2 Coordinación de Investigación, Instituto Jalisciense de Cancerología, Zapopan 45060, Mexico; 3 Departamento de Clínicas Médicas, Instituto de Patología Infecciosa y Experimental, Centro Universitario de Ciencias de la Salud, Universidad de Guadalajara, Guadalajara 44340, Mexico; 4 Campus Zapopan, Universidad del Valle de México (UVM), Zapopan 45010, Mexico; 5 Instituto de Investigación en Odontología, Centro Universitario de Ciencias de la Salud, Universidad de Guadalajara, Guadalajara 44340, Mexico; 6 Doctorado en Microbiología Médica, Centro Universitario de Ciencias de la Salud, Universidad de Guadalajara, Guadalajara 44340, Mexico; 7 Dirección de Desarrollo Institucional, Instituto Jalisciense de Cancerología, Zapopan 45060, Mexico

**Keywords:** chemobrain, chemotherapy-related cognitive impairment, neuroinflammation, gut–brain axis, microbiome, oxidative stress, cancer survivors, quality of life

## Abstract

**Highlights:**

**What are the main findings?**

- The underlying initiators of chemotherapy-related cognitive impairment have not been clearly elucidated. Many mechanisms have been implicated, including neuroinflammation, oxidative stress, mitochondrial dysfunction, blood–brain barrier disruption, impaired neurogenesis, and glial activation.
- The gut–brain axis has been described as a potential chemobrain regulator, where pharmacologically induced dysbiosis, changes in intestinal permeability, neuro-immune signaling, and inflammation may act as modifiers in the development of neurocognitive impairment.

**What are the implications of the main findings?**

- Detection strategies for incipient chemotherapy-related cognitive impairment are key. Combining cognitive screening, biomarkers, neuroimaging, genetic risk profiling, and microbiome analysis is necessary to ensure early intervention.
- Personalized medicine is the most effective intervention strategy to prevent and ameliorate chemotherapy-related cognitive impairment, where pharmacological, nutritional, exercise-based, probiotic, and integrative approaches can help cancer patients and survivors.

**Abstract:**

Chemotherapy is a treatment designed to contain or eradicate neoplastic cells; however, patients may experience various treatment-related adverse effects. Chemotherapy-related cognitive impairment (CRCI), clinically referred to as “chemobrain,” is a frequent complication with a duration ranging from months to years, affecting between 17% and 70% of cancer patients. These cognitive deficits not only impair social, educational, and occupational functioning but may also impact survival outcomes, possibly by interfering with medication adherence and health-related behaviors. Emerging evidence has converged on an integrative cascade in which chemotherapy-induced systemic inflammation, intestinal dysbiosis, blood–brain barrier disruption, microglial/astroglial activation, and impaired hippocampal neurogenesis act in sequence rather than as independent pathways. Underlying pathophysiological mechanisms include neuroinflammation, reduced neurogenesis, loss of dendritic spines, oxidative stress, hormonal changes, epigenetic modifications, and mitochondrial dysfunction. In contrast, repair mechanisms involve complex glial responses, particularly those of astrocytes and microglia. Emerging studies suggest a link between changes in the microbiome and cognitive decline, demonstrating the importance of bidirectional communication in the gut–brain axis. Current research seeks to determine appropriate tests to identify chemobrain. Therefore, several biomarkers, such as GFAP, S100β, and isoprostanes, have been proposed to assess chemobrain, alongside screening tools such as MoCA, MMSE, and CAB-CF, to evaluate cognitive impairment and enable early detection. Pharmacological candidates—including lithium, fluoxetine, methylphenidate, modafinil, metformin, agomelatine, and melatonin—as well as nutritional and lifestyle interventions such as physical exercise, omega-3 fatty acids, curcumin, probiotics, and traditional Chinese medicine formulations—have been investigated, predominantly in animal models. These remain candidate, not validated, therapies; clinical evidence in CRCI populations is limited, heterogeneous, or absent, and well-powered randomized controlled trials are required before any recommendation can be issued. However, optimal strategies for symptom improvement remain unclear, as various approaches have yielded mixed outcomes. This review provides a comprehensive overview of chemobrain, focusing on its molecular mechanisms, interactions with the gut–brain axis, and potential therapeutic targets to improve the quality of life for cancer survivors.

## 1. Introduction

Chemotherapy is a therapeutic modality designed to contain or eradicate neoplastic cells. Although clinical strategies strive to minimize damage to healthy tissues, cytotoxic agents lacking tumor selectivity often damage both malignant and healthy differentiated tissues, resulting in substantial adverse effects [1,2]. First described in the early 1980s by Silberfarb, chemobrain has attracted interest due to increased cancer survivorship, bringing it into sharper clinical focus [3]. Antineoplastic agents can exert direct neurotoxic effects (via multiple pathways) and induce cell death regardless of cell proliferative status [4].

“Chemobrain”—also referred to as “chemotherapy-induced cognitive impairment,” “chemotherapy-related cognitive impairment,” or “chemo fog”—is a frequently used umbrella term that, in this review, refers to the aggregate of cognitive complaints observed in cancer patients before, during, and after chemotherapy. CRCI is now understood to be a multifactorial phenomenon that cannot be attributed solely to the cytotoxic effects of antineoplastic agents. Cancer-related cognitive impairment may reflect any combination of the following three non-mutually exclusive contributors, which are explicitly distinguished throughout this review:

(a) Cancer-associated cognitive impairment present before any treatment, which is driven by tumor biology, baseline systemic inflammation, anemia, cancer-related fatigue, depression, mild cognitive impairment, surgery, endocrine therapies, and the psychological burden of diagnosis.

(b) Chemotherapy-associated cognitive decline, in which cytotoxic agents contribute via direct neurotoxicity, neuroinflammation, oxidative stress, blood–brain barrier disruption, mitochondrial dysfunction, impaired neurogenesis, dendritic spine loss, and epigenetic modifications.

(c) Multimodal treatment-related cognitive impairment, which arises from the combination of chemotherapy with radiotherapy, immunotherapy (immune checkpoint inhibitors, CAR-T cells), endocrine therapy, supportive medications (corticosteroids, antiemetics, hormone replacement), menopause, sleep disturbance, affective symptoms, and pre-existing socioeconomic vulnerabilities.

The present review uses the term “CRCI” in this broader, integrated sense while distinguishing—for each proposed mechanism, biomarker, and intervention—the level of evidence (preclinical vs. clinical; associative vs. mechanistic) and the population in which it was generated [5,6,7]. Overall, studies have reported a prevalence ranging from 17% to 70% among cancer patients, depending on factors such as cancer type, treatment regimen, and assessment tools [6]. Although chemobrain has been mostly studied in breast cancer, it has been demonstrated that around 30% of breast cancer patients might develop this side effect [5]. Half of the cancer patients who manifest it may have long-term effects, persisting for years after treatment [5,6,7]. Depending on the treatment stage, prevalence in patients is around 75% during treatment and around 35% after treatment [5].

Hence, this review aims to provide a comprehensive overview of chemobrain, focusing on its molecular mechanisms, interactions with GBA, and potential therapeutic targets. Thus, we highlight the complexity of chemobrain and the interdisciplinary approaches needed to manage it.

### Literature Search Strategy

A structured yet non-systematic search of PubMed/MEDLINE, Scopus, Web of Science, and Google Scholar from January 2000 to July 2026 informed this narrative review. Boolean search terms were used: “chemobrain,” “chemotherapy-related cognitive impairment,” “CRCI,” “neuroinflammation,” “oxidative stress,” “gut–brain axis,” “microbiome,” “oral dysbiosis,” “biomarkers,” “neurofilament light chain,” “GFAP,” “lithium,” “fluoxetine,” “metformin,” “melatonin,” “curcumin,” “omega-3,” “probiotic,” and “physical exercise.” Systematic reviews and meta-analyses, randomized controlled trials, prospective cohort studies, cross-sectional or case–control clinical studies, and preclinical studies in rodent models or in vitro systems were prioritized when clinical evidence was unavailable. Additional references were manually retrieved from key article reference lists and authoritative reviews. We did not use PRISMA-style selection because this is a narrative review. Selective bias toward mechanistically informative (but mostly preclinical) studies cannot be eliminated.

## 2. Pathophysiological Mechanisms of Chemobrain

### 2.1. Neuronal Damage Induced by Chemotherapy

Several factors influence symptom severity, including the specific chemotherapeutic agent, treatment duration, cancer stage, and the patient’s oncological history [6]. Additionally, host factors such as race, ethnicity, diet, body mass index, socioeconomic status, and gynecological factors (e.g., menopausal status) are hypothesized to influence the onset of manifestations. However, their precise roles warrant further in-depth exploration [5,8,9].

Despite extensive investigation, the specific mechanisms linking diverse chemotherapeutic agents to cognitive deficits in cancer patients remain poorly understood. Some potential causal mechanisms have been proposed, including disruption of cell proliferation and histone modifications in the hippocampus, as well as epigenetic alterations [10], higher concentrations of reactive oxygen species (ROS) leading to increased oxidative stress [11], and hormonal changes [10].

Given its substantial content of polyunsaturated fatty acids, limited antioxidant capacity, and considerable oxygen utilization, the brain exhibits heightened susceptibility to oxidative damage [11]. ROS mediate damage in the brain by affecting essential proteins, lipids, and DNA, ultimately leading to cell death [12].

Chemotherapeutic agents induce various endocrine changes and exert systemic effects on the central nervous system (CNS) (i.e., increased secretion of serotonin, acetylcholine, histamine, and dopamine, which are responsible for anorexia). In addition, it has been shown that chemotherapeutic agents increase leptin, causing appetite to decrease inversely during treatment, ultimately being partially responsible for weight loss [10].

Furthermore, even without chemotherapy, cancer patients maintain a pro-inflammatory state due to cytokine production by tumor cells, such as TNF-α (tumor necrosis factor) and interleukin-6 (IL-6), which are associated not only with increased invasiveness and poor prognosis but also with cognitive impairment and BBB permeability. This leads to a chronic central inflammatory response, resulting in even higher concentrations of these pro-inflammatory cytokines [13].

High concentrations of ROS disrupt the BBB, allowing the entry of pro-inflammatory cytokines synthesized by chemotherapy-targeted tissues; moreover, Toll 4 receptor is activated by oxidative stress, which then releases TNF-α, leading to intracellular activation of nuclear factor kappa-light-chain-enhancer of activated B cells (NF-κB), which then unchains inducible nitric oxide synthase (iNOS), increasing oxidative stress even more, finally resulting in mitochondrial dysfunction and liberation of cytochrome c [13].

Activated NF-κB also increases the proapoptotic proteins Bax and p53 and decreases BCL2 levels. Consequently, the apoptotic cascade is activated, leading to neuronal apoptosis [14,15].

Although protective mechanisms, such as apolipoprotein A1 (ApoA1), exist to mitigate excessive interleukin production—specifically by blocking TNF-α and IL-1β overexpression via the JAK/STAT3 pathway—systemic oxidative stress observed in cancer patients compromises ApoA1 function through oxidation. This functional impairment subsequently disrupts JAK/STAT3 signaling, leading to aberrant NF-κB activation and perpetuating neuronal damage [14,15,16].

**Figure 1:**
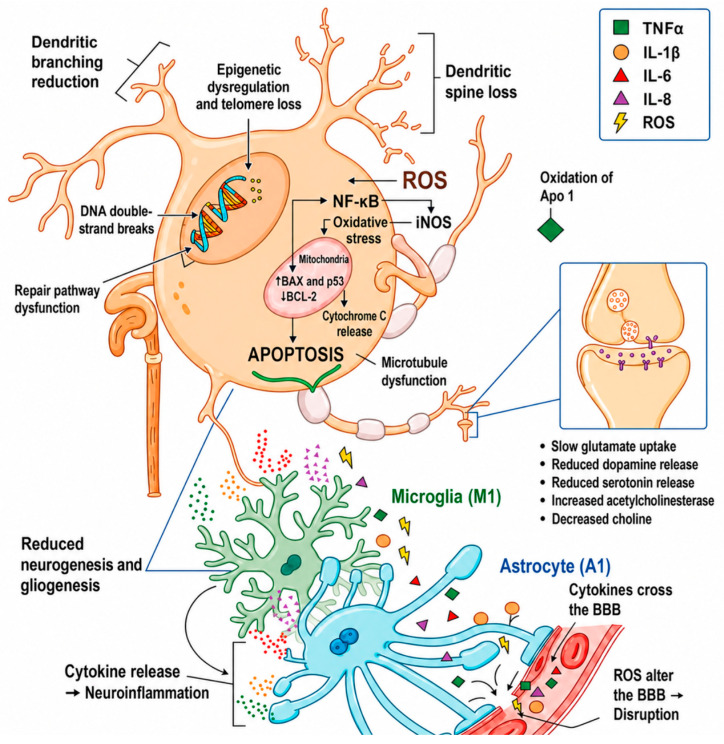
Chemotherapy may initiate (in animal models) a cascade of systemic events culminating in neurotoxic sequelae. The initial phase involves the chemotherapy-induced release of pro-inflammatory cytokines (IL-1β, IL-6, IL-8, and TNFα) and reactive oxygen species (ROS) into systemic circulation. This systemic inflammation disrupts the integrity of the blood–brain barrier (BBB), facilitating the translocation of these mediators into the central nervous system. Within the CNS parenchyma, these factors induce neuroinflammation, characterized by the activation of A1 astrocytes and M1 microglia, thereby amplifying central cytokine release and consequently impairing neurogenesis and gliogenesis. At the neuronal level, increased ROS burden contributes to synaptic spine loss, dendritic retraction, and microtubule destabilization. Dysregulated neurotransmitter uptake and release mechanisms further compromise synaptic function. Nuclear alterations include epigenetic dysregulation and the induction of DNA double-strand breaks, coupled with a downregulation of DNA repair pathways, ultimately promoting apoptosis. ROS promotes the NF-κB pathway, leading to the expression of inducible nitric oxide synthase (iNOS), which, in concert with ROS, exacerbates neuronal oxidative stress. This pro-apoptotic milieu is further evidenced by upregulation of the BAX and P53 signaling pathways, concurrent with downregulation of the anti-apoptotic protein BCL2, culminating in the release of cytochrome C from the mitochondria and subsequent cell death.

The previously discussed mechanisms collectively contribute to cellular damage and death, which underlie the main manifestations of chemobrain in cancer patients. As illustrated in Figure 1, these manifestations include several key neurological changes:

- Reduced neurogenesis: It must be considered that neurogenesis in the adult takes place in the subgranular zone of the dentate gyrus of the hippocampus, the subventricular zone, and the striatum [17,18], and it is common to observe reduced neurogenesis as a result of aging and neurodegenerative diseases, mainly because of a reduction in neural progenitor cells (NPCs) combined with a hostile microenvironment [19].
- Loss of dendritic spines: Dendrites regulate neuroplasticity. They primarily proliferate during the first years of life, stabilizing by adulthood, although dendritic spines remain active, helping to mediate neuroplasticity [20]. Several factors are implicated in the loss of dendritic spines, including age, glutamate toxicity, protein oligomers, and disruption of the cytoskeleton and the ubiquitin-proteasome system [20,21]. The main effect results in cortical thinning, a common finding in cancer patients after chemotherapy [19].
- Abnormal neurotransmitter release: This phenomenon is shown in various neurological disorders, resulting in characteristic clinical features (i.e., a decrease in acetylcholine in Alzheimer disease patients and a decrease in dopamine in Parkinson disease patients) [22]. Specifically in chemobrain, similar effects have been observed; for example, in the treatment with doxorubicin (DOX), a resulting increase in acetylcholinesterase activity, reduction in choline, and slow absorption of glutamate have been reported [23,24,25]; reduction in dopamine release and serotonin in the striatum and raphe nucleus, respectively, after treatment with carboplatin has been implied [26].
- Microtubule deterioration: Microtubules are indispensable structures necessary for the regulation of polarity, axonal transport, signaling, and cell shape. Different chemotherapeutic agents (paclitaxel, docetaxel, ixabepilone, vincristine, and vinblastine) have been associated with microtubule dysregulation [19,27].
- Gliosis: Reactive gliosis, the CNS response to injury, is characterized by glial cell activation and proliferation. Its main purpose is to protect cerebral tissue, limit the propagation of damage, and promote repair; however, in some scenarios, reactive gliosis can be harmful by inhibiting neuronal regeneration [28,29]. In this sense, reactive gliosis has been associated with aberrant reactive gliosis, and the main drugs implicated are methotrexate, docetaxel, and cyclophosphamide [30,31].

### 2.2. Neuroregeneration and Repair Mechanisms

The CNS is composed of various cells, each one with a specific function. These cells can be classified into two categories: neurons and glial cells. Neurons are mostly responsible for transmitting and receiving electrical impulses, while glial cells provide support to neurons [32]. Glial cells comprise four major cell types: microglia, oligodendrocyte precursor cells (OPCs), oligodendrocytes, and astrocytes [33]. Astrocytes are the most abundant glial cells and play an important role in maintaining homeostasis at the synapse, regulating interstitial fluid ions and pH [34,35], as well as in CNS blood flow through the release of arachidonic acid and its derivatives, such as prostaglandins [36,37], They also regulate the uptake and release of neurotransmitters (glutamate, gamma-aminobutyric acid (GABA), and glycine) [37].

In response to CNS trauma, infection, ischemia, or neurodegenerative pathologies, astrocytes transition into a reactive state named “astrocyte reactivity” or “reactive astrogliosis.” This response includes modifications in morphology, functional capacity, and gene expression [38]. In this context, astrocytes are further divided into two phenotypes: A1 astrocytes, which are pro-inflammatory and inhibit axon growth and synapse development, and A2 astrocytes, which exert an anti-inflammatory effect, thereby restoring the BBB and providing neuroprotection [38,39].

Microglia also play an important role in CNS homeostasis; depending on their activation status, they can adopt an M1 phenotype, releasing pro-inflammatory factors (IL-1β and TNF-α), thereby enhancing the cellular response and activating astrocyte reactivity. On the other hand, factors such as IL-4 and IL-10 promote the M2 phenotype, which participates in tissue repair and extracellular matrix reconstruction [39,40].

Chemotherapy treatments such as DOX, mitoxantrone, epirubicin, methotrexate, cyclophosphamide, and paclitaxel induce a higher release of glial fibrillary acidic protein (GFAP), leading to astrogliosis and, consequently, to the expression of the A1 phenotype [41,42,43]. Moreover, therapeutic schemes involving DOX, paclitaxel, and DAC were observed to increase the expression of pro-inflammatory factors [44,45]; a third mechanism was identified in murine models, in which increased glutamate release following treatment with DOX and paclitaxel led to glutamate excitotoxicity and cell death [23]. Microglia also play a key role in the establishment of damage following the administration of chemotherapeutic agents; the expression of the M1 phenotype has been identified [46], enhancing the release of pro-inflammatory agents and thereby promoting even greater expression of the astrocyte A1 phenotype.

**Figure 2:**
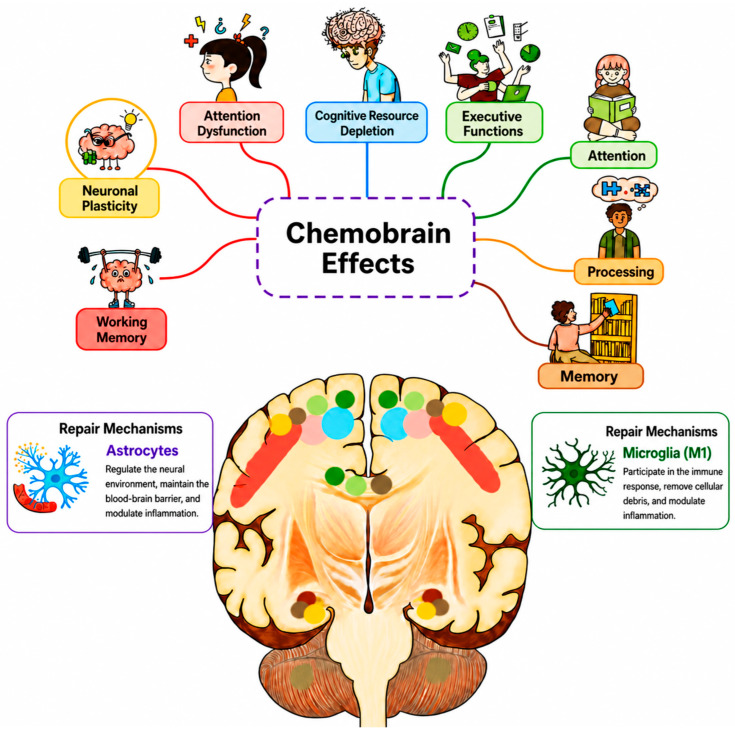
The clinical manifestations of chemotherapy-related cognitive impairment, often termed chemobrain, are multifaceted, with evidence suggesting a correlation between specific symptom domains and their neuroanatomical substrates within the cerebrum and cerebellum. These include deficits in working memory, associated with dysfunction in the prefrontal and parietal cortices; reduced neuronal plasticity observed in the prefrontal cortex and hippocampus; diminished cognitive reserve, primarily linked to the prefrontal cortex; impairments in executive function and attention, implicating the prefrontal cortex and corpus callosum; and slowed neuronal processing across a network encompassing the prefrontal cortex, corpus callosum, hippocampus, and cerebellum. Notably, memory deficits appear to be particularly localized to the hippocampus. Endogenous repair mechanisms in the context of chemotherapy-induced neurotoxicity involve complex glial responses, particularly those of astrocytes and microglia. Astrocytes exhibit a reactive phenotype, characterized by the increased production of both A1 (pro-inflammatory) and A2 (anti-inflammatory) subtypes.

The proposed repair mechanisms involve promoting the early onset of both the astrocyte A2 and microglial M2 phenotypes, which may partially limit cognitive impairment in patients after a chemotherapeutic scheme. To achieve this therapeutic goal, strategies that decrease M1 microglial markers are essential [44,46,47]. As illustrated in Figure 2, these repair mechanisms operate within specific brain regions associated with distinct cognitive functions affected by chemotherapy.

Understanding how these complex neurobiological processes translate into measurable cognitive manifestations is fundamental for the diagnosis and follow-up of patients with chemobrain. The following section presents the main assessment tools used to detect and quantify chemotherapy-associated cognitive impairment.

## 3. Assessment Tools for Cognitive Impairment in Chemobrain

The accurate assessment of chemotherapy-related cognitive impairment requires appropriate screening tools. It is important to note that these instruments do not diagnose specific diseases but rather identify the presence and severity of cognitive impairment, functioning primarily as screening rather than diagnostic tests. Formal diagnosis of cognitive impairment requires meeting established clinical criteria, including comprehensive evaluation of the patient’s clinical background [48]. Here, we focus on cognitive assessment screens commonly used for chemobrain: the Mini-Mental State Examination (MMSE), Montreal Cognitive Assessment (MoCA), and, more recently, the Cognitive Assessment Battery for Chemo Fog Research by CogniFit (CAB-CF) (Table 1).

Cognitive screening tools used in chemobrain research are summarized in Table 1; their methodological properties have been reviewed extensively elsewhere. Tools discussed here are referenced in later sections where specified cognitive endpoints are reported [49,50,51,52,53,54,55,56,57,58,59,60].

## 4. Biomarkers of Neuroinflammation and Cognitive Impairment: Preclinical Evidence, Clinical Findings, and Emerging Hypotheses

### 4.1. Biomarkers for Astrocyte Damage

#### 4.1.1. Glial Fibrillary Acidic Protein (GFAP)

GFAP is a cytoskeletal protein commonly found in astrocytes, which maintains cellular structure and supports cell shape [61]. When brain injury is present, GFAP expression increases due to reactive astrogliosis, in which astroglial cells undergo hypertrophy and release GFAP into the cerebrospinal fluid (CSF) [62]. Several studies have found a relationship between increased CSF GFAP (cGFAP) levels and reduced hippocampal volume, which may reflect loss of synaptic integrity in the same region [63,64]. Moreover, a correlation in cognitive test results and GFAP levels has been demonstrated in neurodegenerative diseases; higher GFAP levels were associated with patients who had a low baseline MMSE score and a rapid cognitive decline, especially in patients with dementia with Lewy bodies [65]. GFAP elevations have been documented in chemotherapy-treated cohorts, but most studies measured astroglial injury rather than cognitive endpoints directly. Its specificity for chemobrain versus general chemotherapy-induced CNS insult has not been established [66,67].

#### 4.1.2. S100 β

S100 β is a calcium-binding protein found mainly in glial cells that helps in regeneration of the central nervous system following neuronal injury [61]. Depending on its concentration, this protein has different effects. When present at nanomolar concentrations (<500 nM), this protein can promote neurite outgrowth and improve neuronal survival during and after injury. In contrast, at micromolar concentrations, it induces the expression of inflammatory cytokines such as IL-6, which in turn induces neuronal apoptosis [68]. Research has mentioned a correlation between increased plasma S100 β levels and cognitive impairment in different diseases, such as Alzheimer’s disease, and in post-chemotherapy cases [68,69]. According to the results, elevated plasma S100 β levels suggest adverse cognitive outcomes in BC patients; however, after adjustment for variables, this association was not significant [68]. S100 β is an emerging candidate for CRCI; however, its independence from confounding variables and specificity for chemotherapy-related cognitive decline require further validation in cognitively phenotyped cohorts.

### 4.2. Biomarkers for Oxidative Stress and Pro-Inflammatory Cytokines

#### 4.2.1. Isoprostanes

Isoprostanes are prostaglandin-like compounds formed by the non-enzymatic peroxidation of arachidonic acid mediated by free radicals [70]. F2-isoprostanes (F2-ISoP) are considered a feasible biomarker of oxidative stress; furthermore, emerging studies have reported a slight association with cognitive decline [71,72]. In Alzheimer’s disease, PGF2α and 8,12-iso-iPF2α VI are associated with neuroinflammation and oxidative stress [73]. On the other hand, in patients with acute lymphocytic leukemia undergoing therapy, higher levels of F2-IsoP were associated with lower scores in cognitive tests [72]. These findings are limited to small samples in pediatric leukemia; the predictive value of F2-IsoP for cognitive trajectories in adult oncology populations remains untested.

#### 4.2.2. Pro-Inflammatory Cytokines

As previously mentioned, chemotherapy induces cytokine production, including IL-1β, IL-6, TNF-α, and IL-8, which can cross the BBB, causing neuroinflammation and cognitive deficits [74]. Prospective studies have shown that increased levels of IL-1β, IL-6, and TNF-α after chemotherapy are associated with reduced cognition in BC patients. In contrast, higher serum IL-8 levels are associated with poorer self-perceived cognition in another BC cohort [75,76]. Additionally, higher serum IL-6 levels were associated with lower MMSE scores in older adults, indicating cognitive decline, potentially related to IL-6-mediated microglial activation [77,78].

Cytokines may help risk-stratify patients at increased risk of persistent cognitive symptoms, but they are nonspecific inflammatory markers and, alone, cannot diagnose chemobrain [79].

### 4.3. Emerging Biomarkers

As research in chemobrain advances, novel biomarkers are being investigated that may offer improved sensitivity, specificity, or practical advantages over traditional biomarkers. These emerging biomarkers represent promising avenues for better monitoring and potentially more targeted interventions for chemobrain.

#### 4.3.1. Exosomes

Findings discussed below (Section 4.3.1, Section 4.3.2 and Section 4.3.3) originate predominantly from neurology literature on Alzheimer disease, mild cognitive impairment, multiple sclerosis, and general aging, rather than from prospective validation in chemotherapy-treated cohorts. Exosomes and neurogranin should therefore be regarded as candidate biomarkers extrapolated to CRCI—not as biomarkers validated in chemobrain populations [62,67,80,81].

Exosomes are 30–200 nm extracellular vesicles with signaling properties that transport proteins, lipids, and genetic material between cells [82]. Secreted by macrophages, dendritic cells, and lymphocytes, exosomes may cross the BBB, representing a potential route through which peripheral inflammation could modulate CNS processes [80]. In addition, exosomes can contain miRNAs, which can disrupt gene expression or initiate neuroinflammation [80]. For instance, Chen et al. [83] identified 12 signature proteins in serum exosomes from patients with taxane-induced peripheral neuropathy.

Although research continues to develop new strategies, exosomes exhibit limitations that require further study to establish them as a feasible biomarker, including the influence of miRNA content, disease-specific signature proteins, and environmental factors on exosome differences [81]. Whether plasma or CSF exosome signatures, neurogranin levels, or neurogranin–CRTH2 interactions can serve as clinically actionable biomarkers in chemotherapy-treated adults remains an open question. No exosome- or neurogranin-based panel has been validated in prospective CRCI cohorts to date.

#### 4.3.2. Neurogranin

Neurogranin is a postsynaptic protein specific to neurons. It has emerged as a biomarker due to its association with neurological processes, including synaptic regeneration and long-term potentiation, via calcium and calmodulin signaling pathways [84]. In animal models, its reduction has been associated with cognitive deficits [84]. This could be explained by synaptic dysfunction and neuronal damage, which are associated with cognitive decline [85]. In chemobrain, synaptic processes can be affected; consequently, an interaction between neurogranin and cognitive function loss might also be detectable. A role for neurogranin in CRCI remains hypothetical; the synaptic mechanisms described here derive from non-chemotherapy models.

#### 4.3.3. Neurofilament Light Chain (NfL)

NfL is a protein expressed in neurons; it is a structural component of axons, and its presence in CSF and blood reflects axonal injury [86]. To date, ultrasensitive techniques such as Simoa (Single Molecule Array) are used to detect extremely low plasma concentrations [87].

Recent findings from Velasco et al. [88] indicate that plasma NfL (pNfL) levels can vary with chemotherapy treatment, treatment stage at evaluation, and mechanisms underlying the cytotoxic effects. In their study, pNfL levels were higher during treatment and decreased after treatment. Overall, pNfL correlated with the severity of chemotherapy-induced peripheral neurotoxicity. Plasma NfL may indicate axonal injury during chemotherapy, though the cited study measured peripheral neurotoxicity rather than central cognitive impairment; application of NfL specifically to CRCI remains to be validated.

The biomarkers discussed above primarily focus on direct indicators of neuronal damage and inflammation (Table 2). However, emerging evidence suggests that systemic factors, particularly those related to the gut microbiome, may play a crucial role in the development and progression of chemobrain [89]. The next section explores the communication between the gut microbiota and the brain and its relevance to chemobrain.

## 5. Chemotherapy-Related Cognitive Impairment and Gut Microbiota: The Gut–Brain Axis

Microbiota refers to the community of organisms within a specific habitat, while the microbiome refers to the interactions between the microbiota and its habitat, including metabolites and genes [90]. The balance in which bacteria and the host benefit from each other is called eubiosis; in contrast, the disruption of the microbiome’s structure and diversity is called dysbiosis, characterized mainly by the loss of dominant bacteria [91].

At birth, microbial colonization begins, which contributes to the development of the immune system. The first flora are facultative anaerobes, such as Enterobacteriaceae and Lactobacilli [92].

Nonetheless, the human gut microbiota changes throughout life; factors such as breastfeeding, childbirth, geography and environment, diet, lifestyle, and age can modify its composition [93]. The crucial phase for gut microbiota development occurs during the first 3 years of life, when the adult microbial composition is fully established [93]. In adulthood, the gut microbiota is composed mainly of four bacterial phyla—Bacteroidota (formerly Bacteroidetes), Actinomycetota (formerly Actinobacteria), Bacillota (formerly Firmicutes), and Pseudomonadota (formerly Proteobacteria)—which together account for over 90% of the adult gut bacterial composition. At the genus level, Bacteroides (phylum Bacteroidota) is among the most prominent taxa in the adult gut and has been reported to decrease following chemotherapy. In contrast, members of Pseudomonadota tend to increase [94,95,96]. A broader classification of the adult microbiome into enterotypes acknowledges inter-individual differences in microbial composition [94].

**Figure 3:**
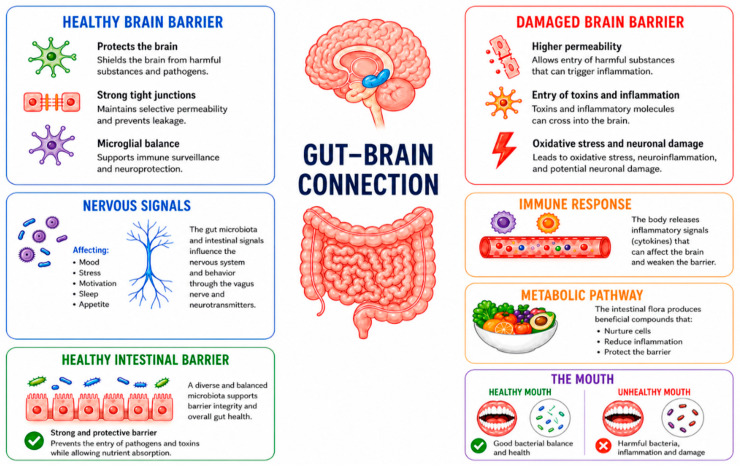
The gut–brain axis constitutes an integrated regulatory system that modulates the permeability and inflammatory status of both the blood–brain barrier (BBB) and the intestinal barrier. A critical feature of chemotherapy-related cognitive impairment (CRCI) is the increased permeability of these barriers, which facilitates the translocation of pro-inflammatory mediators into the central nervous system (CNS). This axis is influenced by multiple interconnected factors, including immune responses, gut microbiota composition, nutritional status, and neural signaling modulated by emotional and psychological stimuli. In this context, dysbiosis of the oral microbiome has been implicated as a driver of chronic low-grade inflammation. Taken together, these factors progressively impair CNS homeostasis, contributing to the neuroinflammatory cascade underlying chemobrain symptomatology.

Recent research has demonstrated a complex, bidirectional interaction between the intestine and the CNS, formerly known as the Gut–Brain Axis (GBA) [97,98]. The gut microbiome can regulate brain activity via the autonomic nervous system, neuroendocrine and immune systems, the hypothalamic–pituitary–adrenal (HPA) axis, and metabolic pathways [99,100] (Figure 3).

One of the main routes of communication between the microbiota and the brain is the neural pathway, especially via the vagus nerve [101]. This nerve acts as a fast channel for transmitting sensory signals from the gut to the brain. Studies have shown that certain strains of *Lactobacillus* and *Bifidobacterium* can modulate vagus nerve activity, thereby affecting the release of neurotransmitters such as GABA, serotonin, and dopamine, which directly influence mood, anxiety, and stress perception [101,102,103,104]. In addition, enteroendocrine cells in the gut can activate vagal receptors by releasing neuroactive peptides such as peptide YY (PYY) and cholecystokinin (CCK), further strengthening this sensory pathway [101].

GBA mainly involves chemical communication, either direct or indirect signaling [100]. Metabolites such as SCFAs, which arise from the breakdown of dietary fiber and amino acids, are thought to play a direct role in the gut–brain axis (GBA) [105]. Acetate, propionate, and butyrate are the most prevalent SCFAs in the intestinal tract; nonetheless, valerate, formate, and caproate are also found in smaller amounts [106]. Once produced in the colon, SCFAs are taken up by intestinal epithelial cells, where they are metabolized and can help reduce intestinal permeability and support immune processes, such as the differentiation, recruitment, and activation of dendritic cells (DCs), macrophages, neutrophils, and monocytes [107]. Accordingly, its mechanisms can modulate the production of cytokines, eicosanoids, and chemokines [108].

As a result, the immune pathway has been recognized as a link in these interactions. Dysbiosis can cause elevated concentrations of pro-inflammatory cytokines, such as IL-6, IL-1β, and TNF-α, that can cross the blood–brain barrier (BBB) or act on nerves, thereby altering neuronal function and contributing to inflammatory processes in the brain [109,110,111]. Additionally, an imbalance can compromise the integrity of the intestinal barrier, allowing lipopolysaccharide (LPS) to enter the bloodstream and induce a systemic inflammatory response that may affect the central nervous system [109]. Furthermore, dysbiosis is linked to cognitive problems such as impaired memory and executive functioning, mood changes, and fatigue [112].

Chemotherapeutic agents can kill or inhibit bacterial growth through cytotoxic effects, thereby altering bacterial communities [113]. Montassier et al. [89] conducted a study in patients with non-Hodgkin’s lymphoma who underwent a myeloablative regimen and high-dose carmustine, etoposide, aracytine, and melphalan for five consecutive days. Outcomes after chemotherapy showed decreases in the Firmicutes and Actinobacteria phyla and in the genera *Ruminococcus, Lachnospira, Clostridium, Collinsella,* and *Bifidobacterium.* In contrast, there was an increase in the abundance of the Proteobacteria phylum and the genera Citrobacter, Klebsiella, Enterococcus, Megasphaera, and Parabacteroides. Moreover, they observed decreased efficiency in energy metabolism, including cofactor, vitamin, and nucleotide metabolism [89].

Another study by Otto-Dobos et al. [114] examined differences in microbiota composition before, during, and after chemotherapy and their relationship to cognitive decline. Their classification was based on a log-ratio analysis of amplicon sequence variants (ASVs) to identify the bacterial taxa whose relative abundances increased (positively associated) or decreased (negatively associated). During chemotherapy, the genera Bacteroides, Collinsella, Escherichia-Shigella, and Eubacterium halli were positively associated. After chemotherapy, the genera *Bacteroides*, *Streptococcus*, *Ruminococcus torques*, *Eggerthalla*, and *Blautia* were positively associated, with many of these taxa continuing to increase beyond the chemotherapy phase. Regarding cognition, increased relative abundance of *Faecalibacterium*, *Fusicatenibacter*, and *Erysipelotrichaceae UCG-003*, and decreased levels of *Bifidobacterium*, *Subdoligranulum*, and *Ruminococcus torques* were observed in patients with a significant decline in memory and verbal learning, both signs associated with chemobrain [5]. In addition, decreased alpha diversity and greater microbial shifts were significantly associated with higher circulating TNF-α levels during chemotherapy.

These changes, commonly termed chemotherapy-induced dysbiosis, have been associated with—and may contribute to—a cascade of inflammatory events that can affect cognitive function through the GBA. However, direct causal relationships in chemotherapy-treated adult humans have not yet been demonstrated [99]. The altered microbial composition leads to decreased metabolite production and increased intestinal permeability [109,110,111]. This allows the “leaky gut” phenomenon to occur, in which other molecules can enter the circulation, activating peripheral immune cells and triggering the release of IL-1β, IL-6, and TNF-α [109,110,111]. Cytokines can reach the CNS via several mechanisms, including crossing the BBB or activating vagal afferents. Once these signals reach the CNS, microglia and astrocytes may be activated—an effect most convincingly documented in rodent models—leading to neuroinflammation in regions critical for cognition, particularly the hippocampus and prefrontal cortex. In animal models, this state has been shown to disrupt synaptic plasticity, neurogenesis, and neurotransmitter systems, producing cognitive impairment analogous to that reported in human CRCI. Whether the same sequence occurs in chemotherapy-treated adult humans, and to what extent, remains an active area of investigation and should not be presented as established causation.

Although the gastrointestinal microbiota has received the most attention in studies of the gut–brain axis, the oral cavity is an important yet often underestimated component of this bidirectional network. The oral cavity is part of the gastrointestinal tract because it is anatomically continuous and chemically connected through saliva, swallowed microorganisms, metabolites, and dietary components. Consequently, oral microbial communities may influence distal gastrointestinal and systemic immune responses [115,116].

Cancer therapies, particularly chemotherapy, profoundly disrupt oral microbial homeostasis [117]. Chemotherapy-induced oral dysbiosis may result from direct cytotoxic injury to the oral epithelium, salivary gland dysfunction, immune suppression, antibiotic exposure, and nutritional alterations secondary to mucositis and dysgeusia [118,119]. These changes promote reductions in microbial diversity and expansion of opportunistic or pro-inflammatory taxa [117]. Importantly, oral complications such as mucositis, xerostomia, and periodontal inflammation may further compromise the epithelial barrier, facilitating microbial translocation and systemic dissemination of inflammatory mediators.

Unquestionably, the oral microbiome is directly associated with dental health and dysbiosis [120,121]. Emerging evidence suggests that oral dysbiosis may contribute to neuroinflammatory pathways involved in chemotherapy-related cognitive impairment (CRCI). Oral pathobionts and their metabolites can access systemic circulation directly through damaged mucosal barriers or indirectly through oral-to-gut microbial translocation [122]. Once established in the gut ecosystem, orally derived microorganisms may exacerbate intestinal dysbiosis, alter gut permeability, and amplify peripheral immune activation [122]. Elevated circulating levels of pro-inflammatory cytokines, including IL-1β, IL-6, and TNF-α, have been implicated in blood–brain barrier dysfunction, microglial activation, and impaired hippocampal neurogenesis, all of which are mechanisms associated with cognitive dysfunction after chemotherapy [123,124].

These same inflammatory mediators are significantly elevated in periodontal disease, one of the most prevalent chronic inflammatory conditions associated with oral dysbiosis [125]. This observation supports the hypothesis that periodontal inflammation may serve as an additional source of systemic immune activation in cancer patients undergoing chemotherapy. Under these conditions, pre-existing or therapy-induced periodontal dysbiosis could exacerbate circulating inflammatory signaling and contribute to neuroinflammatory processes associated with CRCI.

Moreover, periodontal pathogens such as Porphyromonas gingivalis have been associated with neurodegenerative and neuroinflammatory processes, including Alzheimer’s disease, through mechanisms involving lipopolysaccharide (LPS) signaling, systemic inflammation, and amyloidogenic pathways [126,127]. Although direct evidence linking oral dysbiosis to CRCI remains limited, these mechanistic parallels support the hypothesis that the oral microbiome may represent an additional modulator of the gut–brain axis during chemotherapy.

Future studies integrating oral, gut, and neuroimmune profiling may help clarify whether oral microbial signatures could serve as biomarkers or therapeutic targets for preventing cognitive impairment in cancer patients.

Collectively, the data summarized in this section support the concept that chemotherapy-driven dysbiosis and oral microbial imbalance can plausibly contribute to the neuroinflammatory substrate of chemobrain. Still, they have not yet established causation in humans. The cited evidence is largely associative, derived from (i) murine models of chemotherapy-induced dysbiosis, (ii) small cross-sectional clinical cohorts analyzed at a single time point, and (iii) extrapolations from non-oncology neurological populations. In particular, the mechanistic chain of “chemotherapy → dysbiosis → increased intestinal permeability → systemic cytokines → microglial activation → cognitive impairment” remains, for the most part, a working hypothesis supported by indirect links rather than by interventional data in cancer patients. Robust longitudinal studies with serial stool sampling, paired cytokine profiling, gut permeability measurements, and objective cognitive testing are required before microbiome-based interventions can be recommended for this population.

## 6. Chemotherapy-Related Cognitive Impairment and Genetic Variant Associations

**Figure 4:**
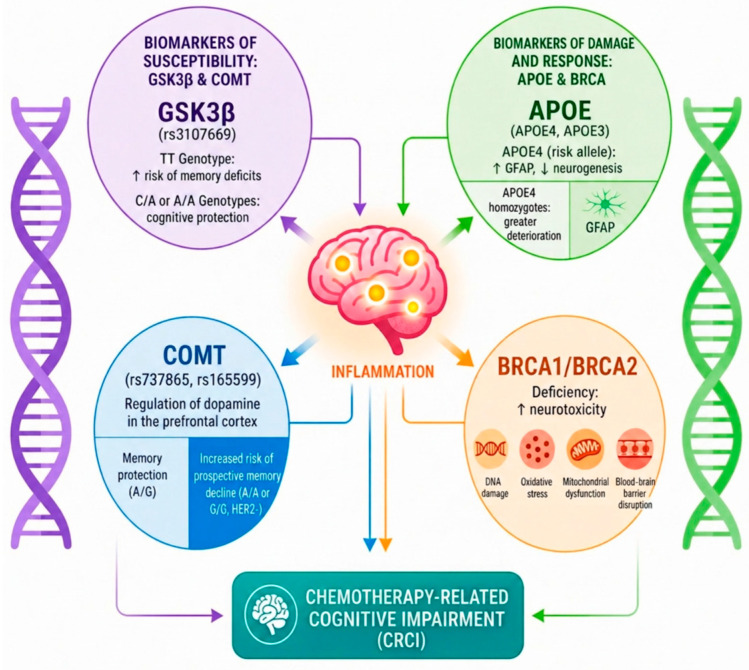
Genes associated with neurocognitive decline exhibit various mechanisms of neuronal damage in individuals with a genetic predisposition. These genes can accelerate cognitive decline in conjunction with chemotherapy-related brain damage. Black arrows pointing upwards indicate an increase and downwards indicate a decrease.

Growing evidence indicates that inter-individual differences in susceptibility to CRCI are partly determined by genetic factors, with single-nucleotide variants (SNVs) and polygenic risk scores linked to specific cognitive domains affected by antineoplastic treatment (Figure 4).

In breast cancer survivors, the GSK3β rs3107669 genetic variant has emerged as a significant risk factor. Patients carrying the C/C genotype exhibited significantly greater retrospective memory deficits after chemotherapy than carriers of the C/A or A/A genotypes, which showed a protective effect on global cognition and memory performance [128]. Bioinformatics analyses further support the involvement of GSK3β in synaptic plasticity and neuronal survival pathways, suggesting a mechanistic link between this variant and chemotherapy-related hippocampal dysfunction.

Similarly, variants in the catechol-O-methyltransferase (COMT) gene consistently associate with prospective memory outcomes, as this gene regulates dopamine levels in the prefrontal cortex. The COMT rs737865 A/G genotype was identified as a protective factor against event-based prospective memory decline, particularly in patients with high tumor proliferation rates (Ki-67 > 14%) [129]. In addition, a separate study focusing on HER2 status demonstrated that COMT rs165599 and rs737865 variants modulate chemotherapy-related prospective memory impairment. Carriers of the rs165599 A/A genotype showed a significantly higher risk of time-based prospective memory decline. In contrast, the rs737865 A/G and G/G genotypes exerted a protective effect on both global cognition and prospective memory performance in patients with HER2− and HER2+ breast cancer [130]. These findings highlight the relevance of dopaminergic pathways in CRCI and suggest that HER2 status may interact with COMT genotype to influence cognitive vulnerability.

Apolipoprotein E (APOE) genotyping has also been implicated in CRCI. In a juvenile rat model, doxorubicin treatment produced significantly greater impairments in visual and spatial memory in rats homozygous for the human APOE4 allele compared with APOE3 rats. These behavioral deficits were accompanied by a marked reduction in hippocampal neurogenesis and elevated serum levels of glial fibrillary acidic protein (GFAP), indicating increased astrocytic damage. These results suggest that the APOE4 genotype increases vulnerability to chemotherapy-related cognitive impairment through impaired neurogenesis [131].

A broader genetic predisposition to low-grade systemic inflammation has also been examined using an inflammation polygenic risk score (iPRS) derived from genome-wide association data in the UK Biobank and validated in a cohort of 802 women with nonmetastatic breast cancer who received chemotherapy (URCC10055/URCC08106 trials). Although the iPRS was not significantly associated with chemotherapy-related cognitive decline (change in FACT-Cog scores: β = −0.36, *p* = 0.871), it showed a significant association with fatigue trajectories (MFSI-SF scores), suggesting that inflammatory genetic burden may differentially influence cognitive versus physical symptoms of chemobrain [132,133].

Finally, genetic variants in *BRCA1* and *BRCA2*—genes critically involved in the homologous recombination repair of DNA double-strand breaks—may significantly exacerbate the neurotoxic effects induced by doxorubicin. In a recent preclinical study, researchers demonstrated that BRCA-deficient neuronal and endothelial cells are markedly more vulnerable to doxorubicin. Because BRCA1/2 proteins are essential for homologous recombination repair, their dysfunction leaves cells unable to repair the DNA double-strand breaks induced by doxorubicin efficiently. This unrepaired damage triggers a cascade of secondary insults: persistent oxidative stress, mitochondrial dysfunction, and disruption of tight-junction proteins that maintain blood–brain barrier integrity. Consequently, *BRCA*-mutant models exhibited greater neuronal loss, elevated reactive oxygen species levels, and increased blood–brain barrier permeability compared with wild-type controls. These findings provide a mechanistic explanation for why patients carrying germline *BRCA* pathogenic variants may experience more severe or prolonged chemotherapy-related cognitive sequelae [134].

Collectively, these findings underscore the genetic heterogeneity of chemobrain and support the development of precision-medicine strategies to ameliorate CRCI.

## 7. Potential Therapeutic and Preventive Interventions for Chemo Brain

Due to the heterogeneous mechanisms underlying chemotherapy-related cognitive impairment, no single treatment approach has emerged as universally effective for prevention or treatment. Instead, a variety of interventions have been developed that may mitigate chemotherapy-related symptoms and improve patients’ quality of life. Among these are pharmacological interventions and nutritional or lifestyle interventions. The therapeutic strategies summarized below span a wide spectrum of putative mechanisms—neurotransmitter modulation, neuroprotection, anti-inflammatory signaling, metabolic regulation, behavioral and lifestyle modification, and gut–brain axis modulation. It is important to acknowledge upfront that the evidence base is uneven: many of the most promising interventions rest entirely on preclinical (rodent or in vitro) studies, whereas clinical evidence, when present, often originates from small pilot trials, single-center cohorts, or post hoc analyses within larger oncology trials. The discussion that follows should therefore be read as an inventory of candidate interventions in various stages of translational development rather than as a list of clinically validated recommendations.

### 7.1. Pharmacological Approaches

Pharmacological interventions for chemobrain target various mechanisms, including neurotransmitter modulation, neuroprotection, anti-inflammatory effects, and metabolic regulation. The following medications have shown potential benefits in preclinical models or clinical studies.

#### 7.1.1. Lithium

Lithium is a mood stabilizer considered a first-line treatment for bipolar disorder type I and maintenance therapy in recurrent unipolar depression [135]. Although the underlying mechanism of action is not fully understood, lithium has been reported to decrease the activity of protein kinase C, which is involved in modifications of neurotransmitters, mainly catecholamines and serotonin [135].

Similarly, Sheng et al. [136] demonstrated that lithium, in combination with prostaglandin E1, can exert neuroprotective effects in cerebral ischemia; thus, this suggests that lithium may act as a potential stimulator of neurogenesis, consistent with other studies [137,138].

Several studies have explored the effects of lithium on cognitive impairment. Nguyen et al. [139] conducted a study in which mice were treated with 20 mg/kg over 8 days. They were randomly assigned into four groups; each group received either saline or 12.8 mg/kg lithium and, after 1 h, another injection with vehicle or 20 mg/kg paclitaxel. Their results demonstrated that lithium and protein kinase C (PKC) inhibitors can prevent CRCI in paclitaxel-treated mice.

In contrast, in a study by Najafi et al. [140], lithium did not show any significant differences in preventing chemotherapy-induced peripheral neuropathy when given in a regimen of four cycles of DOX and cyclophosphamide followed by four cycles of docetaxel. Therefore, it has not been elucidated yet whether lithium could be used as a potential treatment for CRCI in cancer patients.

#### 7.1.2. Fluoxetine

The evidence supporting the discussion of fluoxetine in chemobrain derives predominantly from rodent models of chemotherapy-induced neurotoxicity (e.g., 5-FU, methotrexate, and DOX in rats), where hippocampal-dependent memory, cell proliferation, and inflammatory markers were assessed. Human studies in CRCI populations are sparse and indirect; benefits reported in depression, anxiety, or general cognitive aging cannot be directly translated to chemotherapy-treated cancer patients [51].

Fluoxetine is a phenylpropylamine that is a potent and selective serotonin reuptake inhibitor [141]. Its mechanism of action is based on inhibiting serotonin reuptake in presynaptic serotonin neurons by blocking the reuptake transporter protein, thereby enhancing the effect of this neurotransmitter [140].

Due to the characteristics of this drug, it has been studied as a concomitant alternative to chemotherapy to reduce cognitive impairment. Simultaneous administration of fluoxetine at 10 mg/kg/day for three weeks has been shown to preserve hippocampal-dependent memory and to inhibit the decrease in cell proliferation in rats treated with 5-fluorouracil (5-FU) chemotherapy [141]. Additionally, it has been noted that fluoxetine could be used as a preventive treatment for chemobrain, as it preserves hippocampal cells rather than reversing the effects of 5-FU [142].

In addition, the same 40-day dose, given concurrently with methotrexate in rats, has been reported to reduce CRCI-induced cognitive impairment by counteracting methotrexate’s negative effect on neurogenesis [143].

#### 7.1.3. Methylphenidate

Recognized as a piperidine by-product, it is a psychostimulant used as a primary treatment for attention deficit hyperactivity disorder (ADHD) and narcolepsy [144]. Methylphenidate acts as a monoamine reuptake inhibitor, thereby increasing dopamine and norepinephrine levels in the brain [144]. Nonetheless, some side effects may include insomnia, loss of appetite, and headaches [144,145].

In 1995, Weitzer et al. [146] published a case series where they identified cognitive deficits and behavioral changes in patients with cancer undergoing chemotherapy and radiotherapy treatments. Patients were given methylphenidate doses of 10–20 mg daily, which improved their cognitive performance, suggesting that this treatment could attenuate this side effect.

Furthermore, long-term effects were assessed in a clinical trial involving children who were survivors of leukemia and brain tumors [147]. Doses ranging from 0.3 mg/kg to 0.6 mg/kg were administered over a period of three weeks. The main insights showed improvements in CPT, social skills, attention, and sustained focus.

#### 7.1.4. Modafinil

Modafinil is a psychostimulant prescribed to treat narcolepsy, among other disorders. Its pharmacodynamics are poorly understood, but it appears to have indirect activity as a dopamine receptor agonist and to bind to the noradrenaline transporter, inhibiting its reuptake. It improves the ability to maintain wakefulness, possibly by acting at the level of the hypothalamus, which is involved in regulating the sleep–wake cycle. Some primary studies have reported beneficial effects of methylphenidate and modafinil. Still, the level of evidence could not be determined due to heterogeneity among the included studies or small sample sizes [148].

#### 7.1.5. Metformin

Metformin is a hypoglycemic biguanide used as a first-line treatment for glycemic control in diseases such as type 2 diabetes or polycystic ovary syndrome (PCOS). The effects could be due to the involvement of AMPK pathways activated by metformin. The main known effect of metformin is to inhibit complex I of the mitochondrial respiratory chain moderately. This inhibition results in a deficiency of cellular energy, activating AMPK, which in turn may initiate a cascade of catabolic pathways that generate ATP and deactivate ATP-consuming processes [149,150]. Thus, it decreases hepatic glucose production, enhancing insulin sensitivity. Additionally, metformin may reduce oxidative stress by inhibiting Complex I [151].

Recent research has elucidated how metformin can ameliorate cognitive impairment in neurological diseases such as Alzheimer’s disease and dementia [149,152,153,154]. A similar approach has been observed in animal models treated with cyclophosphamide and cisplatin. Alhowail et al. [155] studied the interaction between metformin and cyclophosphamide in mice, observing improved memory in mice treated with 5 mg/day metformin dissolved in water, compared with those that did not receive it.

Similarly, a study by Zhou et al. [156] confirmed that cisplatin induces peripheral neuropathy, whereas co-administration of metformin completely prevents this adverse effect. At the structural level, cisplatin reduced white matter complexity in the cingulate cortex, a brain region related to cognitive functions. There was also a marked decrease in dendritic spine density and neuronal arborization, both of which are typically associated with neurodegeneration. Metformin was administered at a dose of 100 mg/kg per day, starting 1 day before each cisplatin cycle and continuing for 5 days of cisplatin administration, plus 1 additional day after the last day of the cycle. That is, metformin treatment was pre-emptive and concomitant, not post-treatment, spanning 7 days per cycle.

However, opinions are not fully conclusive, as the effects might differ for each type of chemotherapy; some studies mention that using metformin with DOX treatment does not affect cognitive impairment [157]. In other studies, cognitive impairment in mice has been reported upon initiating concomitant treatment with metformin and DOX, through modulation of IL-1-alpha and IRS-1 expression [158]. Also, the synergistic administration of metformin and fluorouracil exacerbates cognitive impairment in mice and may increase the mortality rate [156].

#### 7.1.6. Agomelatine

Agomelatine and melatonin have been studied in CRCI, primarily in rodent models of chemotherapy-induced neurotoxicity. CRCI-specific randomized clinical evidence in adult cancer patients is lacking. Improvements in mood, sleep, or depression in oncology populations are surrogate endpoints that cannot be assumed to translate into cognitive protection [157,159,160]

Agomelatine is a melatonergic MT1/MT2 receptor agonist and 5-hydroxytryptamine type 2C (5-HT2C) receptor antagonist that has demonstrated significant neuroprotective effects against methotrexate-induced cortical neurotoxicity. In female Wistar rats, oral administration of agomelatine at 20 mg/kg/day for 7 days, initiated before a single intraperitoneal dose of methotrexate (20 mg/kg), effectively attenuated oxidative stress-mediated glial activation by reducing expression of the astrocytic marker glial fibrillary acidic protein (GFAP) and the oligodendrocyte lineage marker oligodendrocyte transcription factor 2 (OLIG2). Agomelatine restored apoptotic homeostasis by normalizing the BAX/BCL2 ratio through downregulation of the proapoptotic protein Bcl-2-associated X protein (BAX) and upregulation of the anti-apoptotic protein B-cell lymphoma 2 (BCL2). These molecular changes were associated with the preservation of the cortical cytoarchitecture and the prevention of neurodegeneration, vascular hyperemia, gliosis, and parenchymal hemorrhage. Agomelatine alone increased BCL2 expression without causing histopathological alterations, supporting its potential as an adjunctive strategy to mitigate chemotherapy-related cortical damage and restore glial homeostasis [159,160].

### 7.2. Nutritional and Lifestyle Interventions

#### 7.2.1. Physical Activity

Physical activity is defined as any movement that involves energy expenditure through skeletal muscle, whereas exercise is characterized as a structured program of movements with a specific purpose [161]. In this context, exercise is primarily considered a therapeutic approach to alleviate the effects of chemobrain, as it has been shown to attenuate the decline in neurogenesis [19].

Physical exercise has been shown to increase VO_2_ peak, alleviate fatigue, enhance quality of life and mood, and improve self-perceived cognitive function [162,163]. Additionally, mitochondrial dysfunction is associated with oxidative stress, thereby affecting brain tissue [164]. As a result, exercise has been described as an alternative to decrease oxidative stress and promote antioxidant capacity [165]. The mechanism underlying exercise effectiveness may involve brain-derived neurotrophic factor (BDNF) expression, a protein associated with brain plasticity and, hence, cognition [166]. Mature BDNF binds to TrkB (tyrosine kinase beta) receptors, activating downstream signaling pathways including PI3K/Akt and MEK/Erk, which promote cell survival [167]. Furthermore, BDNF enhances long-term potentiation (LTP), which can benefit patients during cognitive impairment [168,169].

As a result, studies have elucidated the relationship between exercise interventions and cognitive impairment. Tsai et al. [170] demonstrated that 30 min of exercise, either aerobic or resistance, at an intensity corresponding to 65–75% of the individual target heart rate reserve, improved behavioral performance in decision-making, attention, and reaction time; moreover, it increased BDNF and insulin-like growth factor 1 (IGF-1) serum levels in elderly patients with mild cognitive impairment.

Vaquero et al. [171] did an intervention to assess whether physical activity protects the brain of non-small-cell lung cancer (NSCLC) patients treated with chemotherapy and, small-cell lung cancer (SCLC) patients treated with prophylactic cranial irradiation (PCI), procedures known to induce cognitive impairment. Patients underwent a physical activity program (PAP) consisting of unsupervised walking and supervised cycling sessions, with the frequency and duration increasing over the weeks. The PAP group showed an increase or maintenance of gray matter volume (GMV) in both hippocampi. In SCLC patients, exercise attenuated the volume loss in the right hippocampus. There were no significant differences in cognitive tests, although greater baseline GMV was associated with better maintenance of visuospatial skills.

A systematic review by Ren et al. [172] described that physical exercise, especially aerobic or combined (aerobic and resistance) exercise, significantly improves self-reported cognitive function, cognitive fatigue, and executive function. However, no significant effects were found on processing speed or verbal memory in patients with breast cancer.

Park et al. [173] conducted an experimental study in rats whose cognitive impairment was induced by adjuvant DOX at a dose of 2 mg/kg every 4 weeks. Exercise was aerobic, consisting of low-intensity walking (30 min per day) six days a week for four weeks. The exercise group was observed to increase BDNF and TrkB levels, reduce oxidative stress and cell apoptosis, and improve mitochondrial capacity to handle calcium.

#### 7.2.2. Omega-3 Fatty Acids

Eicosapentaenoic acid (EPA) and docosahexaenoic acid (DHA) are omega-3 fatty acids mainly found in oily fish (such as salmon, tuna, sardines, and mackerel), shellfish, and seafood. Omega-3 fatty acids are integral to the structure of neuronal membranes and to essential processes such as neurotransmission, synaptic plasticity, and neurogenesis [174]. DHA is primarily found in the brain, retinal tissues, and neuronal membranes. It is crucial for the development and maintenance of the nervous system, which is why it is especially important during pregnancy, lactation, and in neurodegenerative diseases [175]. In addition, DHA activates cell survival pathways, such as the Raf and Akt pathways, thereby inhibiting neuronal apoptosis [176]. On the other side, EPA has an anti-inflammatory effect. It is involved in the production of eicosanoids, which regulate processes such as inflammation, immunity, and coagulation [177]. Omega-3 fatty acids can also counteract oxidative stress and improve brain perfusion, both of which are altered after chemotherapy [178].

In breast cancer patients, Omega-3 fatty acids are recognized for their effects in decreasing neuroinflammation and have also been found to reduce depressive and cognitive impairment [179]. Effective doses in this group range from 3 g/day to 7.5 g/day, the latter being the highest [180]. Furthermore, when administered with paclitaxel, omega-3 fatty acids could exert a neuroprotective effect, possibly mediated by reducing pro-inflammatory cytokines and preserving axonal integrity [181].

Van der Meij et al. [182] evaluated the effect of an oral supplementation with omega-3 polyunsaturated fatty acids (2.02 g EPA and 0.92 g DHA daily) on quality of life and functional status in patients with stage III NSCLC undergoing multimodal treatment (chemoradiation therapy). At the end of treatment, the intervention group showed significant improvements in overall health status, physical function, cognitive function, and social function compared with the control group. There was also an increase in daily physical activity and a tendency towards fewer gastrointestinal symptoms. Although CRCI was not directly assessed, significant improvements in cognitive function and quality of life were demonstrated.

In rats, supplementation with Omega-3 polyunsaturated fatty acids (PUFAs) attenuated the effects of DOX [183]. One week before chemotherapy, PUFAs (34% EPA and 24% DHA) were administered daily for three weeks at 1.5 g/kg doses. An improvement was observed in pro-inflammatory cytokines: decreased gene expression of IL-6 and IL-1β in the prefrontal cortex, and reduced expression of TNF-α in the hippocampus. Additionally, the group PUFAs + DOX showed better performance in locomotor and exploratory behavior compared with the DOX group.

Nonetheless, it is important to emphasize the relationship between background diet and supplementation. Interactions with added sugars and PUFAs can attenuate the benefits and reduce their protective effects, and could lead to obesity in some cases [184,185]. Therefore, adequate nutrient intake must be monitored to obtain the expected results.

#### 7.2.3. Curcumin

Curcumin, the principal polyphenolic component of Curcuma longa, has been investigated as a candidate neuroprotective adjunct because of its anti-inflammatory and antioxidant properties [186,187]. In rodent chemobrain models, curcumin decreases TNF-α, IL-6, IL-1β, TGF-β, and MCP-1 expression and downregulates NF-κB and STAT signaling, pathways implicated in cognitive impairment and chronic neuroinflammation [188,189,190,191,192]

Curcumin is the active component from the turmeric plant (Curcuma longa) [193]. Chemically, it is a polyphenol named (1E,6E)-1,7-Bis(4-hydroxy-3-methoxyphenyl)hepta-1,6-diene-3,5-dione; its structure contains two aromatic rings bearing hydroxyl groups, a central conjugated chain with double bonds, and conjugated dicarbonyl groups [194]. Thus, due to its structure, it is considered a highly pleiotropic molecule that has shown anti-inflammatory, neuroprotector, antimicrobial and antioxidant effects [186,187]. Curcumin administration has demonstrated a significant reduction in TNF-α, IL-6, TGF-B and MCP-1 [188,189], and downregulation of transcriptional factors such nuclear factor-κB (NF-κB) and signal transducer and activator of transcription (STAT) proteins, which are linked to cognitive impairment and chronic inflammation [189,190,195]. However, its pharmacokinetics are limited by its rapid metabolism and low oral bioavailability [191,196], and its therapeutic role in chemobrain remains preclinical rather than clinical. A small number of clinical trials—mostly in mixed cancer populations—have reported modest effects on quality of life or generic inflammatory markers. Still, CRCI-specific randomized evidence is sparse and insufficient to support clinical recommendations [197,198]. Reported safe oral doses in selected human trials range from 4 to 12 g/day, although these were not CRCI-specific [199]. Nanocurcumin and phospholipid formulations have been proposed to overcome bioavailability limitations, but most evidence remains in vitro or at the animal level [200,201,202]. Curcumin should presently be regarded as a candidate adjunct in CRCI, not as a validated therapy. Antitumor efficacy considerations, drug interactions (e.g., with irinotecan, tamoxifen), and oncologic compatibility fall outside the scope of this review but should be addressed in any future clinical protocol.

Putri et al. [201] conducted a randomized controlled trial to evaluate the effect of curcumin, in which 78 patients with cervical carcinoma receiving carboplatin-paclitaxel chemotherapy were given curcumin capsules for 14 consecutive days, with 7 days of rest between chemotherapy sessions. The initial dose was 240 mg per day, distributed in 60 mg capsules taken four times a day. Following each cycle of chemotherapy, the dose was increased by 80 mg until a total of 400 mg/day was reached. The results demonstrated improvement in the selective attention test (Stroop), cognitive function with the MoCA Indonesian Version (MoCA-Ina), as well as a decrease in IL-6, GFAP, and isoprostane markers.

Current evidence has demonstrated nanocurcumin’s anticancer effects, mediated by modulation of signaling pathways linked to cancer development, and superior performance over free curcumin; nonetheless, most studies have been conducted in vitro, in animal models, and/or in small samples of clinical trials [202]. Thus, it remains a promising alternative, although further trials are needed to establish the efficacy and long-term safety of nanocurcumin.

#### 7.2.4. Traditional Chinese Medicine

Chinese herbal medicine has been used for centuries. However, it is not yet widely integrated into Western medicine; it focuses on developing customized formulas to address complex conditions, including inflammation, adjuvant chemotherapy, and chemotherapy-related side effects.

The traditional Chinese medicine formula Yifei-sanjie Pill (YFSJ), with patent CN104274518A held by The First Affiliated Hospital of Guangzhou University of Chinese Medicine, consists of eight medicinal herbs in a fixed weight ratio: Sarcandra glabra (15 g), Bombyx batryticatus (10 g), Ranunculus ternatus Thunb. (30 g), Iphigenia indica Kunth (15 g), Fritillaria thunbergii Miq. (15 g), Pinellia ternata (Thunb.) Breit. (15 g), Ganoderma lucidum (Leyss. ex Fr.) Karst. (30 g), and Panax quinquefolius L. (15 g) (Yuan, Y. et al., 2026), which has been widely used as an adjuvant chemotherapy for lung cancer [203,204]; this formula was recently investigated for its ability to mitigate cisplatin (DDP)-induced chemobrain in a subcutaneous Lewis lung carcinoma mouse model. In this randomized preclinical study, tumor-bearing mice received weekly intraperitoneal DDP (5 mg/kg) with or without daily oral YFSJ (4 g/kg) for 28 days; outcomes were assessed through open-field behavioral testing, real-time laser perfusion imaging of cerebral microcirculation, network pharmacology analysis, serum and brain cytokine ELISA, LC-MS quantification of brain DDP content, Western blotting of blood–brain barrier (BBB) and cell-death proteins, and multiple histological techniques. YFSJ significantly attenuated tumor-driven systemic inflammation, preserved BBB integrity, markedly reduced DDP accumulation in brain tissue, restored voluntary locomotor activity and cerebral blood flow, and suppressed both pyroptotic (Caspase-1/GSDMD/IL-1β/IL-18) and ferroptotic (GPX4/GSH/iron/ROS/MDA) neuronal damage, thereby preventing chemotherapy-related cognitive and neurological deficits without directly acting on neurons in vitro [204].

Although traditional Chinese medicine interventions have been rigorously evaluated and shown to be safe and widely used throughout Asia, their integration into Western medical practice remains limited due to cultural and institutional differences. This gap represents a valuable opportunity to explore new therapeutic strategies that combine evidence-based approaches from both medical systems.

#### 7.2.5. Probiotics

The probiotic evidence cited below comes from three non-CRCI tiers that must be distinguished: studies in diabetic rats, scopolamine-induced amnesia, colitis models, and aging rodents; studies in healthy adults, older adults with mild cognitive complaints, or patients with neurodegenerative diseases; and—to date—essentially a single small RCT in breast cancer survivors [205]. Direct extrapolation to CRCI management is not supported by current evidence.

Probiotics are defined as “live microorganisms that, when administered in adequate amounts, confer a health benefit on the host” by the International Scientific Association for Probiotics and Prebiotics [206]. As previously stated, the presence of bacterial colonies leads to the production of SCFAs, which act as mediators in the GBA, maintaining the integrity of the intestinal barrier [105]. Additionally, recent findings suggest that probiotic supplementation can attenuate inflammatory biomarker levels in adults, reducing serum concentrations of TNF-α, IL-6, IL-12, IL-4, and hs-CRP [207]. In this sense, probiotics have shown an important role in human health and in some diseases, including neurocognitive diseases, as they mediate cytoprotective pathways in the epithelium and inhibit ROS and other free radicals [98,208]. Although few clinical trials have examined the effects of probiotic supplementation on chemobrain, many focus on the neuroprotective effects of probiotics.

In animal models, doses of 1 × 10^9^ CFU/rat/day of *Lactobacillus reuteri* GMNL-263 significantly reduced structural changes in the hippocampus in diabetic rats, thereby decreasing cell death and inflammation [209]. Another study by Jang et al. [210] evaluated how colitis-induced changes in the microbiota can lead to memory loss in mice and increase the abundance of Enterobacteriaceae. When colitis was induced, NF-κB was activated, leading to TNF-α expression in the hippocampus of mice; in contrast, when Lactobacillus johnsonii was administered, memory impairment was attenuated. In line with this, Lee et al. showed a relationship between increased BDNF levels and Lactobacillus johnsonii supplementation, leading to improvements in memory impairment. [211] On the other side, *Bifidobacterium* has been implicated in a key role in establishing neural circuits in the mouse hippocampus [212].

In humans, Allen et al. supplemented healthy males with Bifidobacterium longum, and the findings included improved memory and reduced stress [213]. Likewise, older adults showed better cognitive function outcomes with supplementation with Bifidobacterium bifidum BGN4 and Bifidobacterium longum BORI for 12 weeks; furthermore, there was an increase in BDNF compared to the group that did not receive the intervention [214].

Lee et al. [215] conducted a study in which they administered Lactobacillus (L. rhamnosus and L. acidophilus) or a placebo to colorectal cancer survivors for 12 weeks. Their outcomes demonstrated a reduction in depression, anxiety, and fatigue symptoms; additionally, patients showed improved quality of life.

Recent meta-analyses also show that probiotics supplementation can improve cognitive function in neurological diseases such as Alzheimer’s disease and in those with cognitive impairment [216,217]. Moreover, Juan et al. [205] found that probiotic supplementation in patients with chemobrain mitigated these side effects. Nonetheless, to consider probiotics a feasible intervention for chemobrain, further clinical trials are needed.

There is a rationale for using probiotics as a complementary therapy to cancer treatment. However, current evidence is still limited, primarily due to the risk of inducing iatrogenic infection and the lack of robust data on their efficacy, since these patients have an immune system compromised by both the cancer and the treatment; furthermore, due to the frequent use of chemotherapy, radiation therapy, and, especially, antibiotics, the natural protective barriers that prevent colonization by pathogenic microorganisms and the emergence of multidrug-resistant strains may be disrupted. Probiotics, thanks to their properties, can help restore homeostasis and reduce side effects associated with cancer treatment; experimental and clinical evidence suggests that lactic acid bacteria may reduce the toxicity of anticancer therapy, and probiotics are relatively inexpensive and easily accessible [205].

Thus, the administration of probiotics during anticancer therapy is yielding promising clinical results, as it improves intestinal dysbiosis in cancer patients and can significantly improve patient adherence to treatment, as well as their overall quality of life, thereby paving the way for promising ongoing clinical trials in cancer patients undergoing anticancer treatments [205].

#### 7.2.6. Melatonin

The melatonin evidence base in chemobrain relies almost entirely on rat models of cisplatin-, DOX-, 5-FU-, and methotrexate-induced neurotoxicity. Randomized clinical trials in chemotherapy-treated adults with cognitive endpoints are limited, and reported benefits in sleep regulation or general antioxidant status should not be conflated with CRCI efficacy [218].

Melatonin is a hormone endogenously produced by the pineal gland that is widely available as a nutritional supplement in the United States and Canada, where it can be purchased over the counter without a prescription. In contrast, in many other countries melatonin is classified and regulated as a pharmaceutical drug, requiring a medical prescription or stricter oversight for its clinical use. Beyond its well-known role in regulating circadian rhythms, melatonin has attracted considerable interest as a potential neuroprotective agent due to its potent antioxidant, anti-inflammatory, and anti-apoptotic properties. These characteristics have prompted extensive preclinical research evaluating its ability to counteract chemotherapy-induced neurotoxicity.

In a rat model of cisplatin-induced neurotoxicity, intraperitoneal administration of melatonin (20 mg/kg/day for 5 days) significantly improved brain oxidant/antioxidant status and reduced overproduction of pro-inflammatory cytokines such as TNF-α, IL-6, and IL-1β. It restored cognitive and motor performance in T-maze and rotarod tests [218].

Studies using oxaliplatin have further confirmed that pretreatment with melatonin (10 mg/kg) attenuates brain mitochondrial dysfunction, reduces lipid peroxidation and protein carbonyl content, and modulates cytochrome c release and the expression of apoptotic proteins, including Bcl-2 and caspase-3, thereby preserving neuronal integrity and improving muscular strength and thermal nociception [219].

In the context of 5-fluorouracil-induced neurotoxicity, melatonin (8 mg/kg/day for 21 days) protected hippocampal neurogenesis by decreasing the number of p21-positive cells in the subgranular zone of the dentate gyrus, restored the activity of antioxidant enzymes (GPX, CAT, and SOD), reduced malondialdehyde levels, and upregulated Nrf2, DCX, and BDNF protein expression in both the hippocampus and prefrontal cortex [220].

Finally, in a doxorubicin-induced chemobrain model, melatonin (10 mg/kg/day for 30 days) preserved the morphology and complexity of hippocampal microglia and astrocytes, attenuated brain inflammation and oxidative stress, improved mitochondrial function, reduced apoptosis and necroptosis, and restored hippocampal plasticity and cognitive performance in novel object recognition and location tests [157].

Although these findings provide strong preclinical evidence supporting the neuroprotective role of melatonin against chemotherapy-induced neurotoxicity, it must be emphasized that all the studies described above were performed exclusively in rat models. Clinical trials in human patients are still required to determine whether these beneficial effects translate to the clinical setting and to establish the efficacy of melatonin as an adjunctive therapy for chemobrain (Table 3).

## 8. Discussion

The neurological complications associated with cytotoxic chemotherapy have not yet been investigated through a fully integrated and holistic approach. In this review, we synthesize and summarize the available evidence to propose a conceptual framework in which systemic and peripheral inflammation, gut microbiome dysbiosis, blood–brain barrier disruption, glial reactivity, oxidative stress, hormonal dysregulation, mitochondrial dysfunction, epigenetic modifications, impaired neurogenesis, and genetic susceptibility may converge to contribute to chemotherapy-related cognitive impairment [5,12,15,16,74,221,222].

A central conceptual contribution of this review is the integration of the gut–brain axis as a mechanistic amplifier of chemobrain. Chemotherapy-induced dysbiosis alters short-chain fatty acid production, increases intestinal permeability, and favors the translocation of lipopolysaccharide and pro-inflammatory mediators into the systemic circulation [89,99,109,112,114]. On the other hand, very few studies have considered the oral microbiome as a potentially relevant factor contributing to cognitive impairment in patients with cancer. Oral complications are highly common in oncology patients and are often underestimated. However, they may contribute to chronic inflammation, for example, through microbial translocation of oral bacteria from the oral cavity to the gut, which could, in turn, affect the nervous system of patients with cancer [115,116,117,122,125,127].

As part of the multifactorial approach to central nervous system injury associated with cancer or its treatment, this review also addresses genetic factors that provide insights into how cancer-associated genetic variants, such as BRCA1/2, may influence each patient’s systemic inflammatory profile. This raises the possibility that susceptibility to CRCI may be, at least partially, inherited [130,131,132,133].

It is important to note that immunotherapies, targeted therapies, and drug delivery systems all target specific molecular targets. These types of treatments have gradually been added to the list of ways to fight cancer, along with traditional cytotoxic therapy. Drug delivery systems may facilitate the delivery of targeted and cytotoxic therapies across the BBB, with direct implications for the management and long-term sequelae of neurotoxicity observed in oncology patients [223]. Accordingly, the conceptual framework proposed in this review integrates chemotherapy, intestinal dysbiosis, neuroinflammation, increased permeability of the intestine and the BBB, and impaired neurogenesis as interconnected mechanisms that may contribute to chemotherapy-related cognitive impairment.

Chemobrain, or CRCI, remains understudied and subject to considerable uncertainty, with broad research opportunities that warrant attention. Mainly, longitudinal studies that enable observation of the long-term evolution of chemobrain can help identify factors that increase or decrease its duration.

In parallel, one of the main problems is identifying biomarkers predictive of early detection, and even prevention, in patients most susceptible to cognitive impairment. Since chemobrain development involves several complex mechanisms, it is considered unfeasible for a single biomarker to provide sensitivity and specificity for diagnosis and follow-up through interventions. Therefore, multimodal models or approaches must be proposed to help accurately characterize the presence of chemobrain.

Moreover, the potential of combination therapies is essential. Integrating pharmacological, nutritional, physical, and cognitive strategies into a multimodal therapeutic approach could optimize clinical outcomes. Among these strategies, the modulation of the intestinal microbiome is gaining special relevance. Prebiotics, probiotics, and even fecal microbiota transplantation are emerging as novel approaches to prevent or mitigate the effects of chemobrain by recognizing the gut–brain axis as an essential component of neuroimmunomodulation.

Finally, education and awareness among both health professionals and patients are important because promoting early recognition of chemobrain symptoms and providing tools for their management can significantly improve the quality of life of cancer survivors by reducing their worries and feelings of unfitness.

Taken together, these considerations underscore the need for a truly interdisciplinary approach that integrates knowledge from oncology, neurology, psychology, nutrition, and microbiology to provide comprehensive solutions to the complex phenomenon of chemobrain.

This review contributes an integrated and holistic approach to the problem of chemotherapy-related cognitive impairment (CRCI) in several ways:

- It proposes a holistic conceptual framework that considers systemic inflammation, oral and intestinal dysbiosis, glial reactivity, impaired neurogenesis, and susceptibility conferred by genetic variants as interconnected contributors to CRCI.
- It analyzes strategies for detecting and identifying cognitive impairment in the oncological context by reviewing the most commonly used neuropsychological and cognitive assessment tools.
- It reviews pharmacological, nutritional, physical activity-based, and lifestyle interventions that may help reduce the impact of CRCI while placing these approaches within the broader context of future perspectives and emerging knowledge regarding the mechanisms associated with CRCI development.

## 9. Conclusions

### 9.1. Established Findings

Chemobrain, or CRCI, occurs very frequently, is prevalent, and has a strong impact on quality of life and even adherence to cancer treatment; 17 to 70% of cancer patients present it during treatment, and 35% continue with cognitive impairment months or years after finishing cancer therapy. The main affected domains are executive function, working memory, attention, word processing speed, and concentration, which lead to deterioration in daily performance [5,6,224].

The origin of cognitive impairment during oncological treatment is multifactorial; among the factors that converge to produce this deterioration are neuroinflammation, systemic inflammation, disruption of the intestinal epithelial barrier and the BBB, mitochondrial and hormonal dysfunction, epigenetic modifications, impaired neurogenesis with loss of dendritic spines, and aberrant glial reactivity. The gut–brain axis, including the oral microbiome, and each patient’s genetic background play fundamental roles in amplifying cognitive dysfunction. On one hand, short-chain fatty acids (SCFAs), dysbiosis, increased intestinal permeability, translocation of bacterial toxins, and the amplification of systemic inflammation are key mediating mechanisms. Accordingly, understanding and diagnosing CRCI require the use of multiple combined biomarkers and multimodal interventions for its management. Furthermore, the development of specific neuropsychological tools for classifying CRCI by stage and severity remains a critical unmet need in the field [12,19,28,221].

### 9.2. Promising but Unvalidated Mechanisms, Markers, and Candidates

Genetic and epigenetic biomarkers, along with inflammatory molecules, are fundamental for profiling the CRCI. Variants in GSK3β (rs3107669), COMT (rs737865, rs165599), APOE4, and BRCA1/2, as well as polygenic inflammation risk scores, partially explain inter-individual variability in clinical outcomes, providing a basis for risk stratification and personalized interventions [130,131,133]. Biomarker monitoring is clinically feasible; for example, GFAP, S100β, F2-isoprostanes, pro-inflammatory cytokines (IL-1β, IL-6, TNF-α, IL-8), exosomes, neurogranin, and neurofilament light chain constitute a biomarker panel related to systemic and local inflammation and, combined with cognitive screening tools such as MMSE, MoCA, and CAB-CF, enable early detection and objective longitudinal follow-up of CRCI [49,50,58,63,88]. No biomarker panel or screening tool has been validated in chemotherapy-treated cohorts for routine clinical use [62,63,67,88].

### 9.3. Research Priorities and Unresolved Questions

No biomarker panel or pharmacological treatment is currently validated for routine CRCI prevention, diagnosis, or treatment in chemotherapy-treated adult patients. Many questions about the pathophysiology remain unanswered, and most evidence is limited to preclinical or in vitro models. Consequently, numerous gaps remain that must be addressed, particularly the need for prospective and longitudinal studies in humans, including both observational and controlled clinical trials with specific interventions and biomarkers. Most of the available evidence comes from in vitro or preclinical animal models, and longitudinal studies with sufficient methodological rigor and statistical power are still scarce. The inclusion of well-defined and reproducible clinical outcomes, standardized microbiome and biomarker panels, and systematic determination of genetic risk variants into future studies is critical to advance the field of CRCI towards a precision medicine approach for the prevention and management of CRCI.

To enhance patients’ quality of life and mitigate the cognitive effects associated with CRCI, it is essential to address the scarcity of clinical trials and incomplete data.

No patents resulted from the work reported in this manuscript.

## Figures and Tables

**Table 1:** Summary of cognitive screening tools for evaluating cancer-related cognitive impairment.

Feature	MMSE	MoCA	CAB-CF
Full name	Mini-Mental State Examination	Montreal Cognitive Assessment	Cognitive Assessment Battery for Chemo Fog (by CogniFit)
Main purpose	General screening for cognitive impairment	Detection of MCI	Assessment of cognitive effects related to cancer treatments
Cognitive domains assessed	Orientation, memory, attention, and verbal and written language	Memory, visuospatial skills, executive function, attention, language, orientation	Attention, memory, coordination, perception, reasoning
Duration	Less than 15 min	Less than 15 min	30–40 min
Scoring system	Max score: 30 <24: cognitive impairment <18: severe impairment	Max score: 30 <26: MCI ≥26: normal cognition	No fixed scoring ^1^. Provides a risk index and identifies affected domains
Sensitivity/ Specificity	Sensitivity: 79.8–83% Specificity: 73–81.3%	Sensitivity: 90% Specificity: 87%	Not reported
Advantages	Fast, simple to apply, and widely used	Free of charge, training available, high sensitivity for MCI	Digital, easy to use, no prior knowledge needed, good construct validity
Limitations	Biased in highly educated users, limited in executive function, copyrighted since 2001	Requires educational level adjustment, potential educational bias	Long duration, lacks data on sensitivity, specificity, predictive values

MCI: Mild Cognitive Impairment. ^1^ Scores range from 0 to 800 points, though there is no classification system.

**Table 2:** Comparative appraisal of candidate biomarkers for chemotherapy-related cognitive impairment.

Biomarker	Mechanism	Highest Level of Evidence	Citation(s)
GFAP (plasma/CSF)	Astrocytic damage and reactive astrogliosis	Observational clinical studies in neurodegeneration and chemotherapy-induced tauopathy models	[61,62,63,64,65,66,67]
S100β	Glial activation; dual neurotrophic/neurotoxic effect (dose-dependent)	Small cross-sectional cohorts in post-chemotherapy breast cancer patients	[61,68,69]
F_2_-isoprostanes	Systemic lipid peroxidation/oxidative stress	Small clinical studies (predominantly pediatric ALL)	[70,71,72,73]
Pro-inflammatory cytokines (IL-1β, IL-6, TNF-α, IL-8)	Peripheral → central neuroinflammation	Prospective cohorts in breast cancer	[75,76,77,78,79]
Exosomes/miRNAs	Intercellular signaling across the BBB	Preclinical in vivo + small clinical proteomic studies	[80,81,82]
Neurogranin	Post-synaptic integrity/LTP	Preclinical in vivo only	[84]
NFL (plasma)	Axonal injury	Observational cohorts in chemotherapy-induced peripheral neurotoxicity	[86,87,88]

ALL: Acute Lymphocytic Leukemia; BBB: Blood–Brain Barrier; CSF: Cerebrospinal Fluid; GFAP: Glial Fibrillary Acidic Protein; LTP: Long-Term Potentiation.

**Table 3:** Interventions to reduce cognitive impairment: proposed mechanisms of action, levels of evidence, and key limitations.

Intervention	Proposed Mechanism	Highest Evidence Level	Key Limitations
Lithium	PKC inhibition; pro-neurogenesis	Preclinical + one placebo-controlled trial	Negative RCT on neuropathy; no CRCI-specific data
Fluoxetine	Serotonin reuptake inhibition; hippocampal preservation	Preclinical (5-FU, MTX in rats)	No human CRCI data; SSRI–tamoxifen interactions
Methylphenidate	Dopaminergic/noradrenergic enhancement	Case series + small pediatric oncology trials	Small samples; heterogeneous cancer types
Modafinil	Dopaminergic/noradrenergic; wakefulness	Limited observational; meta-analysis of low resolution	No CRCI-specific RCT
Metformin	AMPK activation; antioxidant; mitochondrial protection	Mixed preclinical; paradoxical effects with DOX	Chemotherapy-regimen dependent
Agomelatine	Melatonergic agonism + 5-HT_2_C antagonism; anti-apoptotic	Preclinical only	No CRCI clinical studies
Physical activity	↑ BDNF/IGF-1; antioxidant; anti-inflammatory	Preclinical + several RCTs and systematic reviews in breast and lung cancer	Heterogeneous protocols; modest, inconsistent effect sizes across cognitive domains
Omega-3 (EPA/DHA)	Anti-inflammatory eicosanoids; membrane stabilization	Preclinical + RCTs in NSCLC and breast cancer with mixed cognitive endpoints	Confounded by basal diet; interaction data with chemotherapy limited
Curcumin	NF-κB/STAT inhibition; anti-inflammatory; autophagy modulation	Preclinical + small RCTs in breast and cervical cancer with improvements in selected endpoints	Bioavailability issues; nanocurcumin data still largely preclinical
Yifei-sanjie Pill	Multi-target network pharmacology; BBB protection	Preclinical only	No clinical evidence; cultural/institutional integration barrier
Probiotics	SCFA restoration; TNF-α/IL-6 reduction	Animal models + one small RCT in breast cancer survivors + indirect evidence from Alzheimer meta-analyses	Strain-, dose- and duration-dependent; infection risk in immunosuppressed patients
Melatonin	Antioxidant; anti-inflammatory; anti-apoptotic; mitochondrial protection	Preclinical only	No CRCI RCT; regulatory heterogeneity between countries

BBB: Blood–brain Barrier; CRCI: Chemotherapy-Related Cognitive Impairment; MTX: Methotrexate; NSCLC: Non-small-cell Lung Cancer; RCT: Randomized Controlled Trial; SCFA: Short-chain Fatty Acid; SSRI: Selective Serotonin Reuptake Inhibitor; 5-FU: 5-Fluoracil.

## Data Availability

No new data were created or analyzed during this study. Data sharing does not apply to this article, as it is a review based on previously published literature.
